# Earthquake Magnitude Estimation Using a Total Noise Enhanced Optimization Model

**DOI:** 10.3390/s19061454

**Published:** 2019-03-25

**Authors:** Kyunghyun Lee, Jinhwan Oh, Hyukwoo Lee, Kwanho You

**Affiliations:** Department of Electrical and Computer Engineering, Sungkyunkwan University, Suwon 16419, Korea; naman2001@skku.edu (K.L.); ojinan@skku.edu (J.O.); lhw2065@skku.edu (H.L.)

**Keywords:** heterodyne laser interferometer, total noise enhanced optimization, earthquake magnitude, seismic waves, magnitude estimation

## Abstract

In this paper, a heterodyne laser interferometer, which is used as a sensor for high-precision displacement measurement, is introduced to measure ground vibration and seismic waves as a seismometer. The seismic wave is measured precisely through the displacement variation obtained by the heterodyne laser interferometer. The earthquake magnitude is estimated using only the P-wave magnitudes for the first 3 s through the total noise enhanced optimization (TNEO) model. We use data from southern California to investigate the relationship between peak acceleration amplitude ($P_{d}$) and the earthquake magnitude ($M_{g}$). For precise prediction of the earthquake magnitude using only the $P_{d}$ value, the TNEO model derives the relation equation between $P_{d}$ and the magnitude, considering the noise present in each measured seismic data. The optimal solution is obtained from the TNEO model based objective function. We proved the performance of the proposed method through simulation and experimental results.

## 1. Introduction

In recent years, many efforts have been made to develop an earthquake early warning (EEW) system in various seismic areas around the world. Depending on the characteristics of the EEW station, the early warning time can be up to several tens of seconds. Many methods have been developed to analyze earthquakes, such as spectrum analysis, magnitude measurement, epicenter decision, and intensity interpretation [1,2,3,4]. To predict and reduce the damage caused by an earthquake, the seismic signal needs to be precisely measured and analyzed. As a precision measurement sensor, the heterodyne laser interferometer has many advantages such as wide dynamic range, high accuracy, and sub-nanometer resolution [5,6,7,8,9,10,11]. Using the Doppler effect, the heterodyne laser interferometer detects the shifted frequency of a returned beam from a moving retro-reflector. In addition, the heterodyne laser interferometer can easily be aligned with optical components and manifests a fast response rate, rendering it a mainstay of commercial laser measurement devices. Moreover, the heterodyne laser interferometer has a cost benefit for multi-dimensional installation since the single laser head can measure the seismic signals in multiple paths by dividing the laser source using a beam splitter (BS). The peak acceleration amplitude $P_{d}$ within the first 3 s after the P-wave arrival can be obtained from the precisely measured displacement data using the heterodyne laser interferometer. We determine a relationship between $P_{d}$ and the earthquake magnitude $M_{g}$ for early prediction of the earthquake magnitude.

Olson [12] predicted earthquake magnitude using the evidence of a scaling relationship between the observed parameters $\tau_{p}$ and seismic records of different regions. Kircher [13] used HAZUS technology based on a geographic information analysis system developed by the Federal Emergency Management Agency (FEMA) of the United States. This system analyzes ground motion data using the shakemap algorithm to predict earthquake magnitude and minimize the damage. Wu [14] demonstrated seismic scale prediction using the damping relationship and compared the predicted magnitude with the existing data. The predicted earthquake magnitude value was obtained from the linear relational expression using the received data. Wurman [15] proposed an earthquake alarm system that provides a prediction from the vibration result of the initial seismic center information by analyzing the alert map.

**Figure 1:**
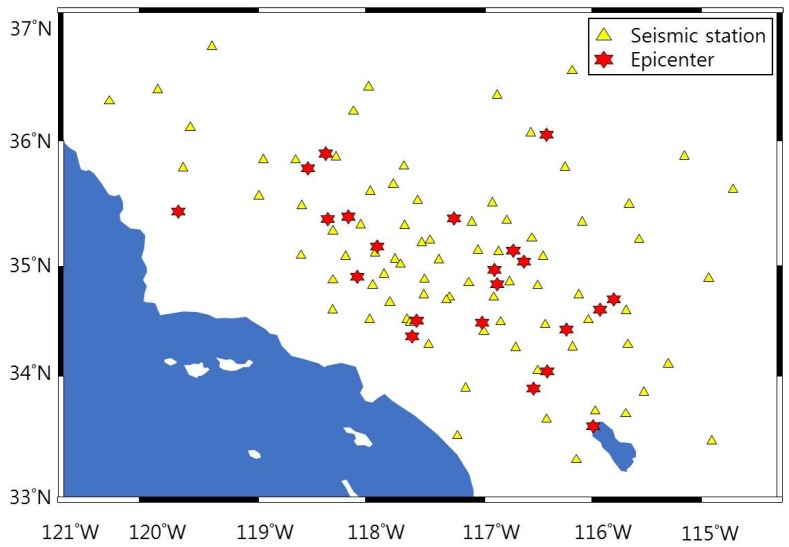
Location of seismic stations of SCSN and the epicenters of events.

In this paper, we propose the total noise enhanced optimization (TNEO) model to estimate earthquake magnitude using the relation between $P_{d}$ and earthquake magnitude $M_{g}$. With a heterodyne laser interferometer as a seismometer, we obtained the seismic wave due to high accuracy and robustness. Using the interferometric intensity signal, we measure more exactly the peak acceleration amplitude ($P_{d}$). The amplitude value of $P_{d}$ is applied to the optimization process of the TNEO model. We build a precision seismometer system using a heterodyne laser interferometer and estimate the earthquake magnitude by measuring the $P_{d}$ value when a new earthquake occurs. Using a laser interferometer and the TNEO model, the earthquake magnitude can be predicted accurately with the minimization of magnitude estimation error. For the formulation of the relation between $P_{d}$ and magnitude, we use seismic records of a regional earthquake from the Southern California Seismic Network (SCSN) operated jointly with the United States Geological Survey (USGS). Figure 1 shows the distributions of the epicenters and the SCSN stations used in this paper.

This paper is organized as follows. Section 2 presents the displacement measurement with the heterodyne laser interferometer system. Section 3 suggests the earthquake magnitude prediction through the TNEO model. Section 4 shows the experimental results of the proposed algorithm and conclusions are provided in Section 5.

## 2. Seismic Wave Measurement Using a Heterodyne Laser Interferometer

The heterodyne laser interferometer is a device that measures the displacement using the optical Doppler effect. The heterodyne laser interferometer used for precision length measurement is applied in the fields of vibration control and semiconductor manufacture, and measures a wide displacement range from sub-nanometer up to several meters. The heterodyne laser interferometer uses two light beams with different frequencies, which are polarized and orthogonal to each other [16,17,18,19,20,21,22]. Two light beams passing through a beam splitter (BS) are emitted from the laser head.

**Figure 2:**
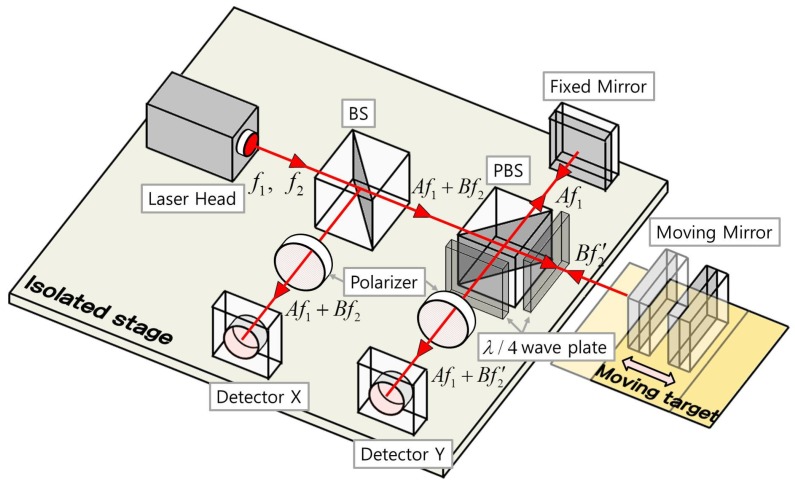
Heterodyne laser interferometer system.

After passing through the BS, one beam is measured from a photo detector and the other beam passes through the polarization beam splitter (PBS). Figure 2 shows a system schematic diagram of the heterodyne laser interferometer. The electric fields emitted by the laser head can be expressed with two frequencies of $f_{1}$ and $f_{2}$ as follows [19]:

(1) $$\begin{array}{ccc} E_{X1} & = & \frac{1}{\sqrt{2}}Ae^{j(2\pi f_{1}t+\Theta_{A})},\\ E_{X2} & = & \frac{1}{\sqrt{2}}Be^{j(2\pi f_{2}t+\Theta_{B})}, \end{array}$$

where $\Theta_{A}$ and $\Theta_{B}$ are the initial phase values and *A* and *B* are the amplitudes of the waves. The photo detector *X* measures the output intensity signal $I_{r}$ as follows:

(2) $$\begin{array}{ccc} I_{r} & \propto & \left(E_{X1}+E_{X2}\right){\left(E_{X1}+E_{X2}\right)}^{*}\\ & = & \frac{1}{2}\left(A^{2}+B^{2}\right)+AB\cos\left[2\pi \Delta ft+(\Theta_{B}-\Theta_{A})\right], \end{array}$$

where Δf refers to the difference between two frequencies (Δf = $f_{1}$−$f_{2}$). Meanwhile, after passing through the PBS, the Doppler shifted frequency happens in the electric fields $E_{Y2}$ as follows:

(3) $$\begin{array}{ccc} E_{Y1} & = & \frac{1}{\sqrt{2}}Ae^{j(2\pi f_{1}t+\Theta_{A})},\\ E_{Y2} & = & \frac{1}{\sqrt{2}}Be^{j(2\pi f_{2}^{\prime }t+\Theta_{B}+\Delta \Theta )}, \end{array}$$

where ΔΘ is the phase difference caused by the Doppler effect in a moving mirror. The photo detector *Y* measures the intensity signal $I_{m}$ reflected from the fixed and moving mirrors as follows:

(4) $$\begin{array}{ccc} I_{m} & \propto & \left(E_{Y1}+E_{Y2}\right){\left(E_{Y1}+E_{Y2}\right)}^{*}\\ & = & \frac{1}{2}\left(A^{2}+B^{2}\right)+AB\cos\left[2\pi \Delta ft+(\Theta_{B}-\Theta_{A})+\Delta \Theta \right]. \end{array}$$

With the results of Equations (2) and (4), two intensity signals ($I_{r}$ and $I_{m}$) are applied to the high pass filter to extract only the AC component that includes the frequency difference and the phase information, respectively:

(5) $$\begin{array}{ccc} I_{r,ac} & \propto & AB\cos(2\pi \Delta ft),\\ I_{m,ac} & \propto & AB\cos(2\pi \Delta ft+\Delta \Theta ). \end{array}$$

To obtain the phase value ΔΘ with high accuracy, a lock-in-amplifier is used to transform into a simplified intensity signal expression. After passing through a low-pass filter, two orthogonal intensity signals ( $I_{x}\propto I_{r,ac}\times I_{m,ac}$ and $I_{y}\propto I_{r,ac}\times I_{m,ac}e^{j\pi /2}$) are derived as follows:

(6) $$\begin{array}{ccc} I_{x} & \propto & \frac{AB}{2}\cos\Delta \Theta ,\\ I_{y} & \propto & \frac{AB}{2}\sin\Delta \Theta . \end{array}$$

For the ideal situation, the phase of ΔΘ can be found as a trigonometric function using $I_{x}$ and $I_{y}$.

(7) $$\begin{array}{c} \Delta \Theta =\tan^{-1}\left(\frac{I_{y}}{I_{x}}\right). \end{array}$$

The phase of ΔΘ shows the displacement according to the following equation:

(8) $$\begin{array}{c} \Delta D=\frac{\Delta \Theta \lambda }{4\pi n}, \end{array}$$

where *n* is the refractive index of air, λ is the average wavelength of $f_{1}$ and $f_{2}$, ΔD is the displacement variation, and ΔΘ is obtained from Equation (7).

## 3. Earthquake Magnitude Estimation Using a TNEO Model

Using the earthquake magnitude data and $P_{d}$ values obtained from the past seismic data, we can derive a linear relational expression by applying the TNEO model. The optimization model that signifies the estimation error between the predicted magnitude and a real earthquake magnitude can be derived with the linear relational expression [23,24]. When a new earthquake is detected at the observatory, we predict the earthquake magnitude using the $P_{d}$ value, which is measured during the first 3 s after the P-wave arrival, through the minimization of the optimization model.

We set up a linear regression model based on $P_{d}$, the earthquake magnitude $M_{g}$, and the epicentral distance *R* in km for a single earthquake [14,25]:

(9) $$\begin{array}{c} \log\left(P_{d}\right)=k_{1}\cdot M_{g}+k_{2}\cdot \log\left(R\right)+k_{3}, \end{array}$$

where $k_{1}$, $k_{2}$, and $k_{3}$ are constant values to be determined for the regression analysis. With *N* earthquake data in the real environment, Equation (9) can be rewritten in the following matrix form:

(10) $$\begin{array}{c} P=Mx+e, \end{array}$$

where

$$\begin{array}{ccc} P & = & \left[\begin{array}{c} \log(P_{d,1})\\ \vdots \\ \log(P_{d,N}) \end{array}\right],\;\;x=\left[\begin{array}{c} k_{1}\\ k_{2}\\ k_{3} \end{array}\right],\;\;M=\left[m\;\;s\;\;o\right], \end{array}$$

$$\begin{array}{ccc} m & = & \left[\begin{array}{c} M_{g,1}\\ \vdots \\ M_{g,N} \end{array}\right],\;\;s=\left[\begin{array}{c} \log(R_{1})\\ \vdots \\ \log(R_{N}) \end{array}\right],\;\;o=\left[\begin{array}{c} 1\\ \vdots \\ 1 \end{array}\right]. \end{array}$$

e means the error due to the environmental noise factors that occur in the real environment. Since the measurement of a natural seismic signal can be contaminated by non-uniform environmental factors, the seismic measurement data include errors [26,27,28,29,30]. In an ideal case in which the variables P and M are free of noise factors, the following equation can be satisfied:

(11) $$\begin{array}{c} P_{0}=M_{0}x. \end{array}$$

The *i*-th peak acceleration amplitude $P_{d,i}$ containing the environmental error can be expressed as

(12) $$\begin{array}{c} \log\left(P_{d,i}+\Delta P_{d,i}\right)=\log P_{d,i}+\log\left(1+\frac{\Delta P_{d,i}}{P_{d,i}}\right), \end{array}$$

where $\Delta P_{d,i}$ denotes the error value. Assuming the error value is small enough to satisfy $\Delta P_{d,i}\ll P_{d,i}$, it can be approximated as $\log\left(1+\Delta P_{d,i}/P_{d,i}\right)\approx \Delta P_{d,i}/P_{d,i}$ by using the Taylor series, and Equation (12) is rewritten as follows:

(13) $$\begin{array}{c} \log\left(P_{d,i}+\Delta P_{d,i}\right)\approx \log P_{d,i}+\frac{\Delta P_{d,i}}{P_{d,i}}. \end{array}$$

Assuming matrix M also includes an error value, the estimation error of Equation (10) due to the total noise N = $\left[n_{a}\;\;n_{b}\;\;n_{c}\;\;\Delta P_{d}\right]$ from the measurements of M and P can be derived as follows [31]:

(14) $$\begin{array}{c} Mx-P=\Delta Mx-\Delta P=GN, \end{array}$$

where

$$\begin{array}{ccc} G & = & \left[G_{1}\;\;G_{2}\;\;G_{3}\;\;G_{4}\right],\\ G_{i} & = & \left[\begin{array}{cccc} k_{i} & 0 & \cdots & 0\\ 0 & k_{i} & \cdots & 0\\ \vdots & \vdots & \ddots & \vdots \\ 0 & 0 & \cdots & k_{i} \end{array}\right],\;\;i\in \left\{1,2,3\right\},\\ G_{4} & = & \left[\begin{array}{cccc} -\frac{1}{P_{d,1}} & 0 & \cdots & 0\\ 0 & -\frac{1}{P_{d,2}} & \cdots & 0\\ \vdots & \vdots & \ddots & \vdots \\ 0 & 0 & \cdots & -\frac{1}{P_{d,N}} \end{array}\right]. \end{array}$$

In Equation (14), $n_{a}$, $n_{b}$, $n_{c}$, and $\Delta P_{d}$ denote ${\left[n_{a,1}\;\cdots \;n_{a,N}\right]}^{T}$, ${\left[n_{b,1}\;\cdots \;n_{b,N}\right]}^{T}$, ${\left[n_{c,1}\;\cdots \;n_{c,N}\right]}^{T}$, and ${\left[\Delta P_{d,1}\;\cdots \;\Delta P_{d,N}\right]}^{T}$, respectively. To minimize the estimation of error due to the measurement noise, the objective function needs to be derived using the amount of measurement noise. Using the pseudoinverse method, the amount of noise N can be formulated from Equation (14) as follows:

(15) $$\begin{array}{c} N=G^{†}\left(Mx-P\right)={\left(G^{T}G\right)}^{-1}G^{T}\left(Mx-P\right). \end{array}$$

From Equation (15), the objective function that represents the amount of measurement noise can be acquired as the following equation:

(16) $$\begin{array}{c} F\left(x\right)=N^{T}N={\left(Mx-P\right)}^{T}{\left(GG^{T}\right)}^{-1}\left(Mx-P\right). \end{array}$$

The optimal parameter of x needs to be obtained to minimize the measurement noise. The parameter value x of the optimized linear regression model that predicts the earthquake magnitude using the initial seismic information can be determined by simply solving Equation (16) via the least squares technique without additional constraints:

(17) $$\begin{array}{ccc} x^{*} & = & \arg\min_{x}{\left(Mx-P\right)}^{T}{\left(GG^{T}\right)}^{-1}\left(Mx-P\right)\\ & = & {\left[M^{T}{\left(GG^{T}\right)}^{-1}M\right]}^{-1}M^{T}{\left(GG^{T}\right)}^{-1}P. \end{array}$$

Equation (17) is the optimal solution of a TNEO model. The linear regression model based magnitude prediction algorithm using the $P_{d}$ value, denoted by Equation (9), can be formulated with the substitution of the optimized parameters $x^{*}$ = ${\left[k_{1}^{*}\;\;k_{2}^{*}\;\;k_{3}^{*}\right]}^{T}$.

## 4. Simulation and Experimental Results

In this section, we verify the performance of the proposed magnitude estimation using a heterodyne laser interferometer and compare the magnitude errors between the standard least squares method and the TNEO model based optimal solution. In our experiments, the seismic signal generated using a linear stage is measured by a heterodyne laser interferometer. The arrival time of the P- and S-waves of the earthquake is precisely determined using the measured seismic data, since the $P_{d}$ value is observed as a maximum acceleration amplitude of an earthquake during the first 3 s after the P-wave arrival. Moreover, the epicentral distance is calculated using the arrival times of the P- and S-waves.

For the arrival time of each wave, the short-time Fourier transform (STFT) [32] and instantaneous frequency (IF) estimation [19,33] are applied to the intensity signal ($I_{y}$) data. As we estimate the earthquake magnitude within a restricted area, the propagation velocities of waves are supposed to be known from the recorded seismic data. The predicted magnitude of the earthquake using the measured $P_{d}$ value through the proposed regression model is compared with the results of the standard least squares method for the performance confirmation in our experiments.

The experiment uses a heterodyne laser interferometer with a He-Ne laser head (Wavetronics: WT-307B). To generate the seismic signal, we use a linear stage driven by a 2-phase stepping motor (Sciencetown: PSA6520) with a 20 mm stroke. Using the linear stage, seismic wave movement is generated such that the artificial movement represents the natural characteristics of an earthquake. We set the mean wavelength ($\lambda_{m}$) from the laser head as 632.9 nm and the air refractive index (*n*) as 1.000000026. To demonstrate the validity of the experiment, the seismic records of a regional earthquake from the SCSN of the USGS are used as seismic reference data for the TNEO based linear regression model. The seismic records were collected from earthquakes that had occurred naturally in the southern California area for the past ten years. With 200 seismic wave records, the relation parameters $x^{*}$ = ${\left[k_{1}^{*}\;\;k_{2}^{*}\;\;k_{3}^{*}\right]}^{T}$ that minimize the amount of measurement noise, denoted by Equation (16), are obtained from Equation (17). The best-fitting regression model between the earthquake magnitude $M_{g}$ and $P_{d}$ is expressed as follows:

(18) $$\begin{array}{c} \log\left(P_{d}\right)=1.0084\cdot M_{g}-0.4555\cdot \log\left(R\right)+4.4805. \end{array}$$

In our experiments, the earthquake magnitude is estimated using the $P_{d}$ value via the optimized regression model in Equation (18).

**Figure 3:**
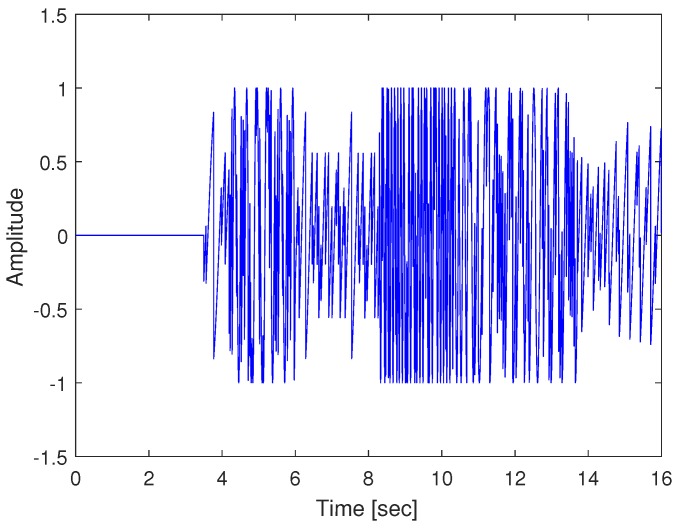
Laser interferometric intensity signal ($I_{y}$) for spectrum analysis.

**Figure 4:**
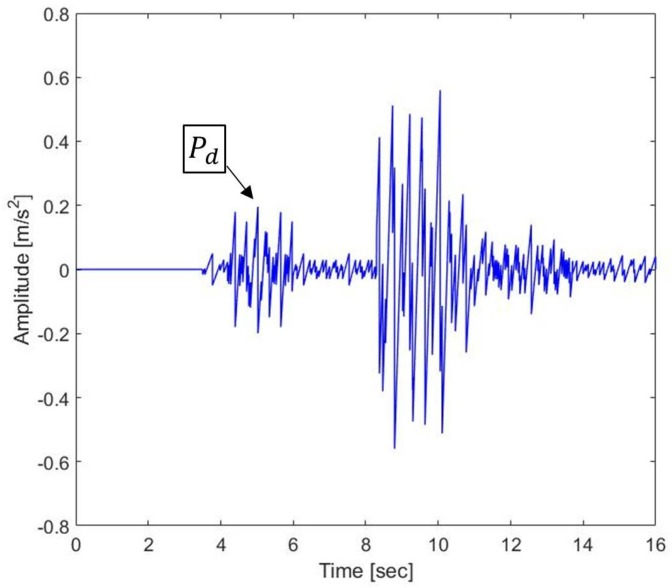
Detection of peak acceleration amplitude ($P_{d}$) using a heterodyne laser interferometer.

Figure 3 shows the laser interferometric intensity signal ($I_{y}$) for spectrum analysis with AB = 2 in Equation (6). The arrival time of the P- and S-waves can be determined by the extremely densified intensities of $I_{y}$, since the density of $I_{y}$ is proportional to the acceleration amplitude of the seismic wave measurements. The extremely densified points of $I_{y}$ in Figure 3 can be interpreted as the arrival time of the P- and S-waves at 3.5 and 8.2 s, respectively. Thus, the epicentral distance is computed with the P-S time of 4.7 s. Figure 4 shows the acceleration of the seismic wave movement measured by a laser interferometer. The $P_{d}$ value is the maximum acceleration value from 3.5 to 6.5 s since we confirmed that the arrival time of the P-wave is 3.5 s. In Figure 4, the $P_{d}$ value is 0.199 m/s${}^{2}$ at 4.824 s. The magnitude of the earthquake can be calculated using the obtained epicentral distance and the $P_{d}$ value through the TNEO based regression model denoted by Equation (18).

**Figure 5:**
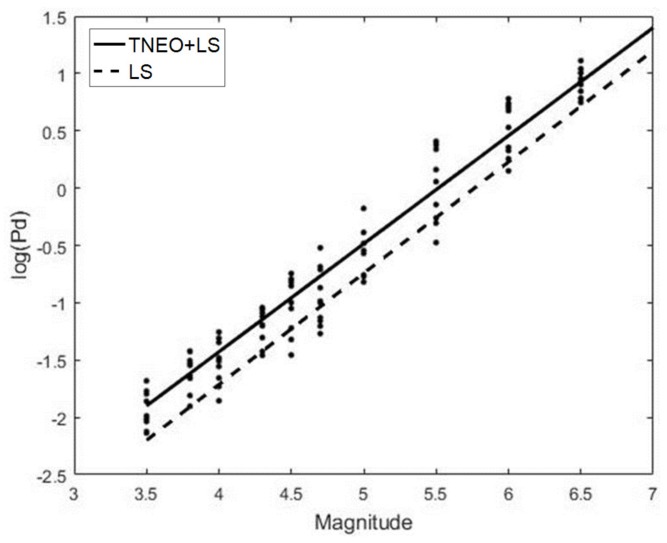
Linear regression model comparison for $P_{d}$-$M_{g}$ relation.

**Figure 6:**
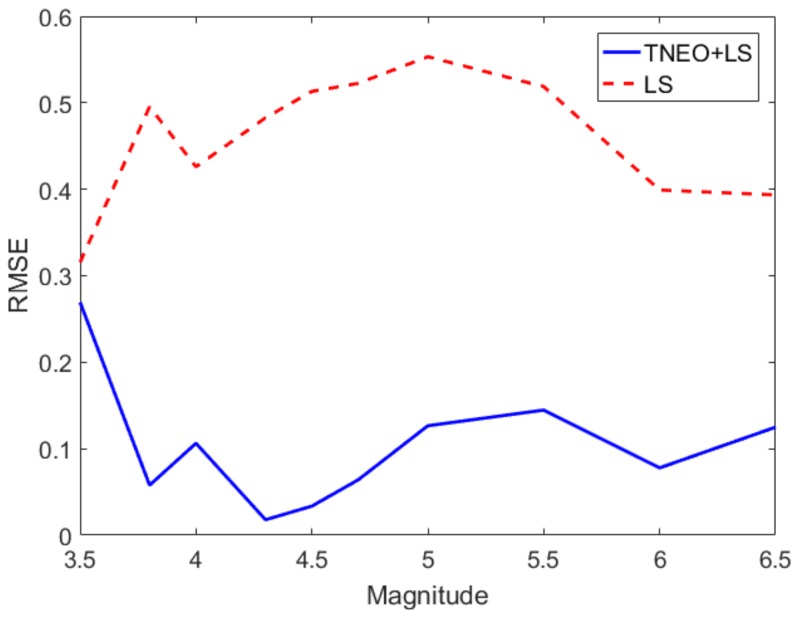
RMSE change for earthquake magnitude.

Figure 5 shows the results of the relation derivation with the standard least squares (LS) algorithm and TNEO regression model based on the data. In Figure 5, the dots refer to the seismic records that are used as reference data from USGS. The solid line and the dashed line denote the estimated relationship between $P_{d}$ and the magnitude using TNEO and the LS algorithm, respectively. The average error value of $\log(P_{d})$ for each magnitude using the LS method and the TNEO model are 0.5945 and 0.2659, respectively. The magnitude prediction algorithm based on the TNEO model becomes more effective than the standard LS algorithm. Figure 6 shows the root mean square error (RMSE) of the magnitude estimation using the TNEO model and LS method. The dashed line represents the RMSE of the estimation using the LS method and the solid line denotes the result of the proposed TNEO model. As shown in Figure 6, the estimation using the LS algorithm shows a higher average RMSE value than that using the TNEO model at each interval.

**Figure 7:**
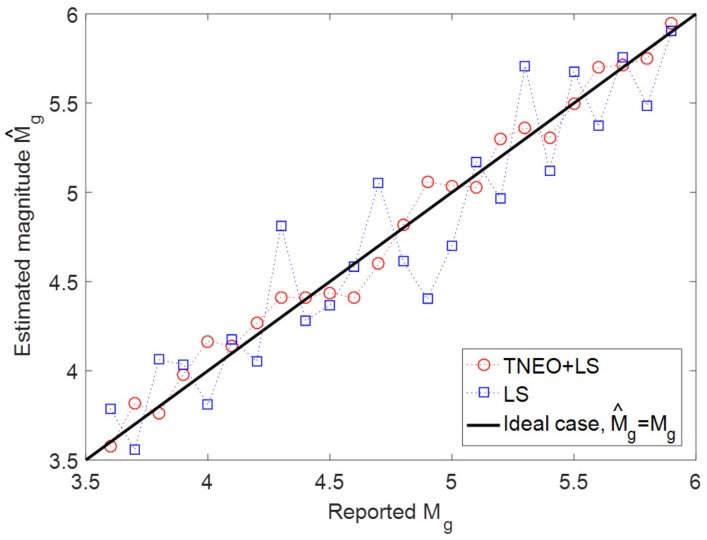
Average magnitude comparison between the TNEO model and general model.

In Figure 7, magnitudes of 200 separate events have been estimated using an LS solution based on the TNEO model (Equation (14)) and the LS solution based on the general model (Equation (10)), respectively. As a solution for both models, the least squares algorithm is applied with the same conditions. To compare the estimation performance, each data group under the same magnitude is used for a separate estimation process. In Figure 7, the circle marker and the rectangular marker denote the average of the estimated magnitude per each data group using TNEO model and LS model, respectively. For an ideal case, ${\hat{M}}_{g}$ should be equal to $M_{g}$. As shown in Figure 7, the result of TNEO model estimation is relatively close to the ideal case compared with the general LS estimation.

## 5. Conclusions

In this paper, we proposed an adaptive estimation algorithm to predict earthquake magnitude accurately using a TNEO regression model. The seismic movement obtained by a laser interferometer was used to measure the maximum amplitude of the P-wave within the first 3 s after the P-wave arrives. Moreover, the precisely measured P-S time using the laser interferometer was used for the computation of the epicentral distance. The TNEO model obtained the coefficients of regression scheme that minimize the prediction error between the real magnitude and the estimated magnitude using the $P_{d}$ value. The estimation parameters of the regression model that represent the relation between the earthquake magnitude and the $P_{d}$ value obtained the optimal solution of the magnitude prediction process. We showed that the TNEO model based regression algorithm is superior to the LS method in predicting an earthquake’s magnitude. The effectiveness of the proposed earthquake magnitude estimation based on a laser interferometric seismometer was shown through simulation and experimental results.
